# BiGAN: LncRNA-disease association prediction based on bidirectional generative adversarial network

**DOI:** 10.1186/s12859-021-04273-7

**Published:** 2021-06-30

**Authors:** Qiang Yang, Xiaokun Li

**Affiliations:** 1 School of Electronic Engineering, Heilongjiang University, Harbin, 150080 China; 2 Postdoctoral Program of Heilongjiang Hengxun Technology Co., Ltd., Harbin, 150090 China

**Keywords:** LncRNA-disease association, LncRNA sequence similarity, Disease semantic similarity, Bidirectional generative adversarial network

## Abstract

**Background:**

An increasing number of studies have shown that lncRNAs are crucial for the control of hormones and the regulation of various physiological processes in the human body, and deletion mutations in RNA are related to many human diseases. LncRNA- disease association prediction is very useful for understanding pathogenesis, diagnosis, and prevention of diseases, and is helpful for labelling relevant biological information.

**Results:**

In this manuscript, we propose a computational model named bidirectional generative adversarial network (BiGAN), which consists of an encoder, a generator, and a discriminator to predict new lncRNA-disease associations. We construct features between lncRNA and disease pairs by utilizing the disease semantic similarity, lncRNA sequence similarity, and Gaussian interaction profile kernel similarities of lncRNAs and diseases. The BiGAN maps the latent features of similarity features to predict unverified association between lncRNAs and diseases. The computational results have proved that the BiGAN performs significantly better than other state-of-the-art approaches in cross-validation. We employed the proposed model to predict candidate lncRNAs for renal cancer and colon cancer. The results are promising. Case studies show that almost 70% of lncRNAs in the top 10 prediction lists are verified by recent biological research.

**Conclusion:**

The experimental results indicated that our proposed model had an accurate predictive ability for the association of lncRNA-disease pairs.

## Background

Conventional molecular biology assumes genetic information is stored primarily in the sequences of genes that code for proteins [1]. However, an increasing number of studies have revealed that protein-coding genes account for only a tiny fraction of human genome (approximately 1.5%), while the other human genes are not involved in the protein-coding sequence [2–5]. In addition, in recent years, an increasing amount of experimental evidence has demonstrated that in most biological processes non-coding RNAs (ncRNAs) are involved extensively [6, 7]. In particular, long non-coding RNAs (lncRNAs) with a nucleotide sequence length greater than 200 are large and essential non-coding RNAs [8, 9]. Recently, with the improvement of computational power and experimental techniques, thousands of lncRNAs have been discovered, from lower eukaryotes such as paramecia to humans [10]. Although lncRNAs cannot encode proteins, they play important roles in biochemical reactions in the human body, such as protein translation, expression, gene regulation, immune regulation, oncogenesis and tissue development [11]. Currently, there are many accumulated peices of evidence that the association between lncRNAs and diseases is particularly important. Many diseases caused by lncRNAs are complex and difficult to control, such as prostate cancer, colon cancer, Alzheimer’s disease, cardiovascular disease, and lung cancer [12–16]. For instance, the oncogenic effect of lncRNA-H19 can be inhibited by the under-regulation of renal carcinoma cells [17]. Therefore, it is essential to predict lncRNA-disease associations. It can help us to understand the biological processes and the molecular mechanisms of human diseases from the perspective of ncRNAs.

In recent years, a large number of computational methods have been proposed to predict lncRNA-disease associations for application in biological experimental verification. These approaches are mainly divided into three categories. The first predicts the correlation between unknown lncRNAs and diseases by sorting out disease similarities, lncRNA similarities, and the association between lncRNAs and diseases based on a random walk. However, if there is no known interaction of relevant lncRNA information on the new disease or no known interaction of relevant disease information on the new lncRNA, it is difficult for these methods to be applied to the relevant association prediction. For example, Sun et al. [18]., restarted the random walk and applyied it to the functional similarity network of lncRNAs. They proposed a computational model called RWRLNCD to detect the associations between diseases and lncRNAs in humans. In addition, Yao et al. [19]., Zhou et al. [20]., also raised a similar calculation approach based on a random walk. However, they focused more on the construction of a heterogeneous network to achieve the purpose of disease association prediction forzz lncRNAs.

The second method utilizes semi-supervised learning methods and machine learning models to extract the feature space between the known lncRNA-disease association and predict the unverified association of the two. In 2013, Chen et al. [22]., created a semi-supervised learning model based on the Laplacian regularized least-squares method (LRLS). In 2016, Lan et al. [21]., blended different data sources and employed a classifier SVM to predict potential interactions between diseases and lncRNAs. Their model solved the problem that the method of LRLS for predicting lncRNA-disease associations was degraded by using two combined classifiers. However, the method proposed by Lan et al. still had great deficiencies in the effective fusion of different lncRNA cores. In 2019, Li et al. [23] proposed a disease gene prioritization based on graph convolutional neural network (PGCN). Their method empolyed end-to-end manner to embedding the hetergeneous network of diseases and genes.

The third category constructs a correlation matrix of lncRNA-disease pairs based on known experimental data. The sequence similarity between lncRNAs and semantic similarity between diseases are integrated to find their associations with genes, to obtain the potential association between lncRNAs and diseases. Such an approach, however, relies heavily on extensive genetic records. As a result, these models are greatly limited in their predictive tasks. For example, in 2012, Chen et al. [24]., predicted lncRNA-disease associations based on the close relationship between genes through the association between diseases and lncRNA genes. However, the identification of lncRNA position characteristics is still a tough task. In 2015, a computational model called KATZ was proposed by Chen et al. [25]. The main idea of KATZ is to integrate disease semantic similarities and the expression profile of lncRNAs. However, the low expression level of lncRNA inhibited the function of this model.

In the past decade, deep learning has become one of the most popular subjects in scientific research. Many deep learning models have been created by scholars and applied in various fields. At the same time, great success has been achieved in the field of biology. In particular, computational models based on neural networks have made outstanding contributions to the task of prediction [26]. As a neural network, the auto-encoder can learn input data through unsupervised learning, strongly represent potential features, effectively reduce sample noise, and randomly generate data similar to the training data [27].

Therefore, this paper proposed a bidirectional generative adversarial network that uses an encoder and generator to learn high-level features in latent space, and a discriminator to predict lncRNA-disease associations.

## Results

### Parameter settings

In our study, the input length of the BiGAN encoder is 6137 and the output length is 100. The lengths of the generator input and output are opposite to those of the encoder input and output. We applied a fully connected layer and ReLU activation function on each network and employed a cross-entropy function as the loss function. Adam was also used to optimize our model. The number of epochs was set as 5, and the batch size was set as 64 in our predicted model.

### Evaluation metrics

To evaluate the predictive ability of the BiGAN on the association between lncRNA and disease pairs, we validated our proposed model using five-fold and 10-fold cross-validation. Almost all samples were taken as candidates in each cross-validation. Therefore, the distribution closest to the original samples makes the evaluation results highly reliable. We utilized the experimentally verified lncRNA-disease associations as samples, while all the unverified associations of the lncRNA-disease pair were used as candidates. Hence, we could rank each candidate sample based on the predicted score. In the rank list, a threshold was given. Samples with lncRNA- disease association prediction scores above the threshold were considered true positive (TP). For each given threshold, we can find the corresponding TP to determine the true positive ratio (TPR), which is also known as sensitivity. Similarly, we can obtain the false negative (FN) samples among the candidate samples by setting a threshold, and the corresponding false positive ratio (FPR) is also called the 1-specificity. The TPR and the FPR can be calculated as follows:

1 $$\begin{aligned} FPR = \frac{FP}{FP+TN}, TPR = \frac{TP}{TP+FN} \end{aligned}$$

where TP is the number of positive samples, and FN is the number of negative samples whose prediction scores are higher than the threshold but considered as a negative sample. TN is the number of negative samples, and FP is the number of negative samples whose prediction scores are lower than the threshold but considered positive samples.

**Fig. 1:**
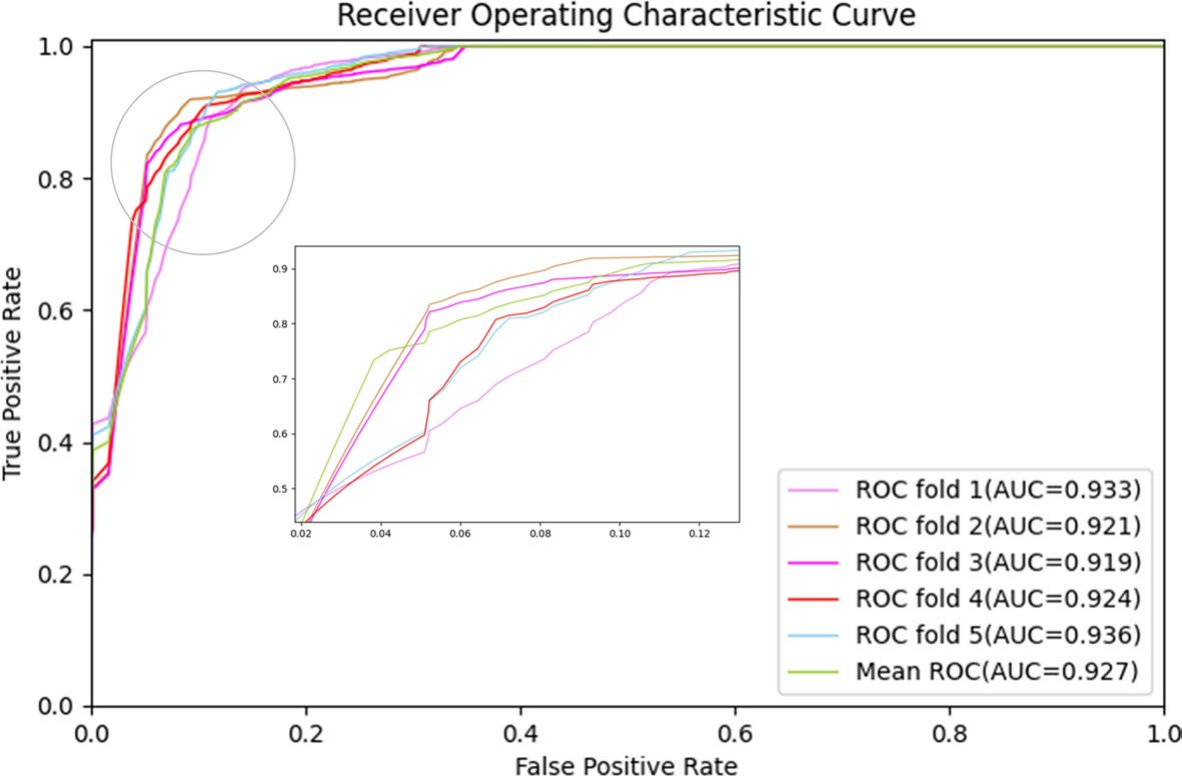
ROC curves of the BiGAN in five-fold cross-validation in LncRNADisease dataset

TPR and FPR denote the proportion of the number of lncRNA-disease association prediction scores over or under a given threshold in the test samples respectively. Therefore, according to the different thresholds, we can plot the receiver operating characteristic (ROC) curve, which is shown in Fig. 1. At the same time, we calculated the area under the ROC curve(AUC) to evaluate the lncRNA-disease association ability of our proposed model [28]. The higher the AUC value is, the better the performance of the BiGAN. When the AUC value of reaches 1, it is considered that the BiGAN can perfectly predict lncRNA- disease associations. When the value is close to 0.5, it is considered to predict the association randomly. To balance the samples of known lncRNA-disease associations and unknown lncRNA-disease associations, we also utilized the precision-recall (PR) curve to estimate our BiGAN [29]. Precision and Recall are defined as follows:

2 $$\begin{aligned} Precision = \frac{TP}{TP+FP},Recall = \frac{TP}{TP+FN} \end{aligned}$$

In addition, we utilized statistical parameters to measure the performance of our predicted model, such as the F1-score, accuracy, and Matthews Correlation Coefficient (MCC). The experimental results in the three datasets are shown in Table 1.

**Table 1:** Ten-fold cross-validation results performed by the BiGAN on three datasets

Dataset	AUC	AUPR	Accuracy	F1-score	MCC
MNDR	0.929	0.901	0.967	0.874	0.867
LncRNADisease	0.931	0.911	0.961	0.871	0.864
LncRNACancer	0.934	0.905	0.979	0.873	0.864

### Comparison with other methods

We compared the BiGAN with other four other advanced methods to prove that our model can predict the associations of lncRNA-disease pairs effectively. The three datasets mentioned above were employed as gold standard training sets to evaluate the other methods. The four methods were the probabilistic prediction model of the association between lncRNAs and disease based on Naive Bayes Classifier [30], the Convolutional Neural Network based on an attention mechanism for the lncRNA genes relationship with diseases (CNNLDA) [31], identifying known lncRNA-disease association by using Topological Information (TILDA) [32], and the web service for discovering lncRNA-disease interaction through mixing multiple biological data resources (LDAP).

**Fig. 2:**
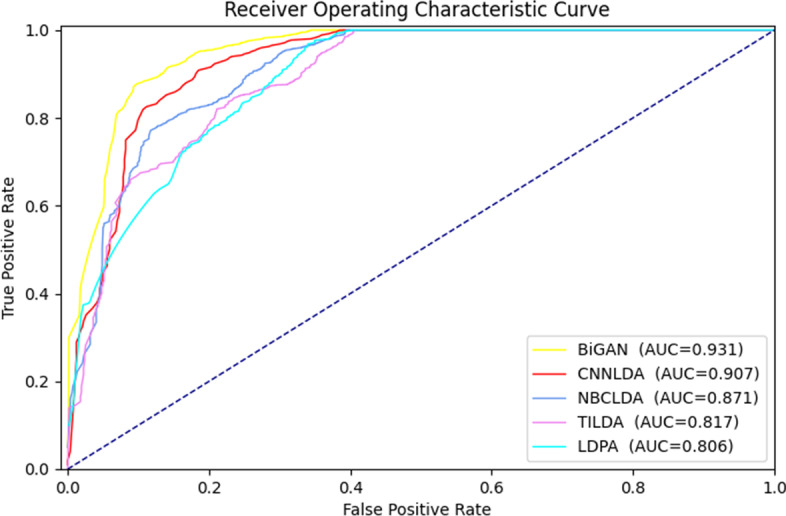
ROC curves in 10-fold cross-validation by different methods

As shown in Fig. 2, the AUC (0.931) of the BiGAN is the highest compared to those of the other methods in 10-fold cross-validation on the LncRNADisease dataset. The AUC values of CNNLDA, NBCLDA, TILDA, and LDAP are 0.907, 0.871, 0.817, and 0.806, respectively. The AUC and AUPR values for all methods in five-fold cross-validation are shown in Table 2.

**Table 2:** The AUC and AUPR values for all methods in five-fold cross-validation

Five-fold cross-validation	BiGAN	CNNLDA	NBCLDA	TILDA	LDAP
AUC	0.927	0.914	0.821	0.815	0.776
AUPR	0.917	0.897	0.807	0.796	0.753

**Fig. 3:**
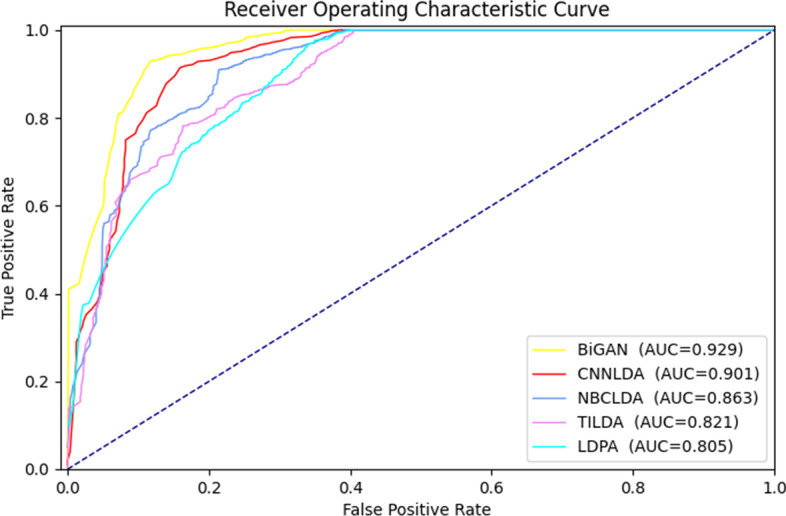
Ten-fold cross-validation ROC curves obtained by different methods on the Lnc2Cancer dataset

**Fig. 4:**
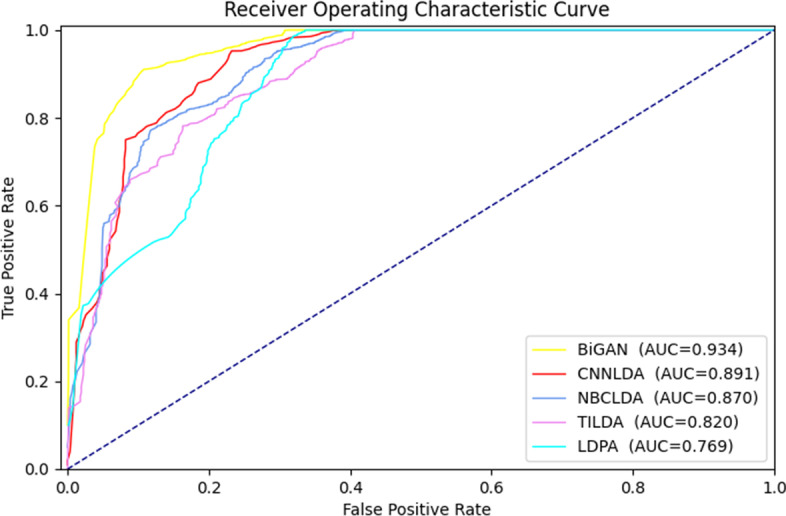
Ten-fold cross-validation ROC curves obtained by different methods on the MNDR dataset

To verify that our prediction model performs well not only in a single dataset, but also in two other datasets. As shown in Fig. 3a, the area enclosed by the ROC curve and the coordinate axis of BiGAN and different models in the dataset Lnc2Cancer in 10-fold cross-validation. In the Lnc2Cancer dataset, except for the fact that the AUC value of TILDA was slightly higher than that for LncRNADisease, the AUC values of the models was lower. Most likely, this is because the underlying cancer was more difficult to predict than a common disease. So the performance of most models on Lnc2Cancer dataset was poorer. In the MNDR, the AUC of the BiGAN reached the highest value, 0.934 (Fig. 4). The results in the three datasets demonstrated that the proposed model was not solely capable of efficient prediction of lncRNA-disease association in a specific dataset. As the results show, the BiGAN has strong robustness and generalization ability, which is better than other state-of-the-art models in most datasets.

### Case studies on colon cancer and renal cancer

To demonstrate the ability of the BiGAN to predict the latent association between lncRNAs and diseases, we measured our method based on case studies on the Lnc2Cancer dataset and MNDR dataset.

Colon cancer is one of the most dangerous cancers and one of the main causes of death among humans. The relationship among the codes in the sequence of lncRNAs associated with colon cancer is that these sequences may cause cancer. With the development of cancer research, lncRNAs had become an essential target for colon cancer prevention, diagnosis, and treatment. In the Lnc2Cancer and MNDR datasets, we applied the BiGAN to predict the associations between colon cancer and lncRNAs, and 7 experimentally verified lncRNAs were on the top ten prediction list. ANRIL can suppress the expression of other RNAs in the late phase of the DNA damage response to repair DNA to normal levels [33]. Experimental results show that the control network composed of UCA1 and other RNAs is a potential factor in the treatment of colon cancer [34]. Additionally, the migration ability of colon cancer cells is significantly inhibited and blocked when TUG1 is expressed, and the overexpression of TUG1 may accelerate the cell migration process of colon cancer cells [35].More details are shown in Table 3.

**Table 3:** Top ten predicted results between colon cancer and renal cancer by the BiGAN with experimental validation in the literature on Lnc2Cancer dataset

Colon cancer			Ṟenal cancer		
Name of lncRNAs	Rank	Pubmed ID	Name of lncRNAs	Rank	Pubmed ID
ANRIL	3	23416462	UCA1	5	31996265
CCAT1	6	31039730	MALAT1	1	31250518
H19	4	31602323	ACTN4	6	Unknown
ENST	8	Unknown	PVT1	3	30105850
XIAP-AS1	9	30892955	FAL1	9	Unknown
P14AS	7	Unknown	HOTAIR	2	30105850
GAS5	2	28722800	H19	7	29214011
UCA1	5	30652355	RAB31	10	Unknown
TUG1	1	27634385	NBAT1	4	31298469
DANCR	10	Unknown	MEG3	8	31071531

More than 250 thousand new cases of renal cancer are diagnosed each year, and renal cancer is recognized as one of the top ten common cancers. It is important to find an association between renal cancer progression and the dysregulation of certain lncRNAs. Among all the lncRNA candidates predicted by the BiGAN as being associated with renal cancer, 7 lncRNAs were among the top 10 in the predicted list, (MALAT1 1st, HOTAIR 2nd, PVT1 3rd, NBAT1 4th, UCA1 5th, H19 6th, and MEG3 7th). MALAT1 reduces the expression of miR-203 to promote the expression of BIRC5 and accelerate the occurrence and development of renal cell carcinoma [36]. The long non-coding RNA HOTAIR accelerates $\alpha$-2, 8-salivary transferases in renal cell carcinoma malignancies by wetting pre-miniaturized microRNA-124 [37]. By down-regulating miR-16-5p, lncRNA PVT1 promoted the invasion, proliferation, and epithelial-mesenchymal transformation of renal cell carcinoma cells [38].

According to the above description, the BiGAN can achieve good performance in predicting unknown association between lncRNA-disease pairs. Therefore, our approach can be widely used for predicting unverified lncRNA-disease associations recorded in the databases. All candidate associations are prioritized and the predicted results can be used for future research and experimental validation.

## Discussion

In the experiment, we integrated the comprehensive similarity vectors of known lncRNA-disease correlations to present their relationship as the first step. Then, the BiGAN model was built to predict the unverified associations between lncRNAs and diseases by learning high-level features in latent space from the similarity vectors. Although the BiGAN seems to outperform than other advanced methods in the above evaluation, it still has some room for improvement.

Based on an auto-encoder, the BiGAN can automatically recognize the comprehensive similarity characteristics of lncRNAs and diseases, eliminate noise, and reduce dimensions. It always learns the annotated biological patterns perfectly. However, we found that our proposed model did not achieve the best performance in predicting the association between lncRNAs and diseases. In our research, two main factors may affect the results. On the one hand, the performance of the BiGAN strongly depends on the similarity eigenvectors which are computed through handcrafted measurements. However, it is not easy to extract the similarity features from high-dimensional data by using these methods. On the other hand, the structure of the BiGAN is based on an auto-encoder whose main idea is to compress the features into low dimensions and learn the latent representation. Thus, we assume that the BiGAN cannot share and propagate information perfectly in each network layer.

In this study, we did not further consider whether the performance would be impacted by the values of the parameters that were set as default. In fact, the parameter settings are significantly important to a certain model because suitable parameters can help the model learn privileged information from the eigenvectors, particularly for complex associated features. In recent years, heteroscedastic dropout has been one of the best regularization techniques for controlling deep neural networks to absorb privileged information. Thus, we will take what has been discussed above as our future work to improve the prediction ability of lncRNA-disease associations.

## Conclusions

In this manuscript, we introduce an unsupervised learning lncRNA- disease association prediction framework called BiGAN. The model includes three main parts, a feature extractor based on similarity algorithm, a bidirectional generator based on autoencoder, and a discriminator that jointly discriminates data and latent space features. We integrated lncRNA sequence similarity, disease semantic similarity, and Gaussian interaction profile kernel similarity to mine the high-level representation of the potential space between lncRNAs and diseases. Ultimately, the BiGAN can effectively predict the associations between lncRNAs based on the latent relationship of the integrated similarity vectors. In 10-fold cross-validation and five-fold cross-validation, our AUC values were 0.931 and 0.927, respectively, indicating the effectiveness of our prediction model. We also compared our model with other state-of-the-art methods, and the results revealed that the BiGAN was superior to other advanced methods. Additionally, we conducted case studies on colon cancer and renal cancer. The case results showed that our proposed model had an accurate predictive ability for the association of lncRNA-disease pairs.

## Methods

### Datasets

To better train our BiGAN model, we collected three experimentally validated datasets from MNDR v3.0, Lnc2Cancer, and LncRNADisease. Below is a brief description of the datasets used.

The first dataset is from the mammalian ncRNA disease repository (MNDR) with coverage and annotation proposed by Lin et al. 24 August 2020 [39]. We extracted association information about human lncRNA-disease pairs in MNDR, consisting of two datasets. One of the databases is experimentally verified association information, covering 742 human diseases, 25,494 human lncRNAs, and 39,783 lncRNA-disease associations, which can be used as a training set. The other dataset is the association information of predicted lncRNA-disease pairs, covering 231 human diseases, 17,713 human lncRNAs, and 52,144 pieces of association information, which can be used as a validation set.

The second dataset was released on 8 January 2019, and contains experimentally validated lncRNA-disease correlations downloaded from LncRNADisease V2.0 [40]. We also collected another special ncRNA dataset named circRNA whose sequences were sufficiently long (>200) in this dataset. After removing the lncRNA disease pairs that were not labelled with IDs and that lacked features, we deleted duplicate samples describing the lncRNA-disease relationship according to known experimental evidence. From this, we obtained 205,959 interaction associations for 529 human diseases and 19,166 lncRNAs. In addition, 823 circRNAs and 529 human diseases, and 1004 interaction associations were included. This dataset contains more comprehensive information than the other two datasets.

The third dataset was published on 30 June 2020, and contains experimentally proven lncRNA-disease correlations which were based on the Lnc2Cancer V3.0 dataset [41]. After removing the lncRNA disease pairs that were not labelled with IDs and that lacked characteristics, we deleted duplicate samples describing the lncRNA-disease relationships according to known experimental evidence. As a result, 216 human diseases,2659 human lncRNAs, and 9254 human lncRNA-disease interaction associations were obtained. Compared with lnc2cancer2.0 published in 2018, the number of diseases have increased by 51 and the number of lncRNAs increased by more than 1000, and the lncRNA-disease association nearly doubled. This allows us to collect enough data to learn the features of the latent space between lncRNAs and diseases in training the BiGAN.

#### LncRNA-disease association

**Fig. 5:**
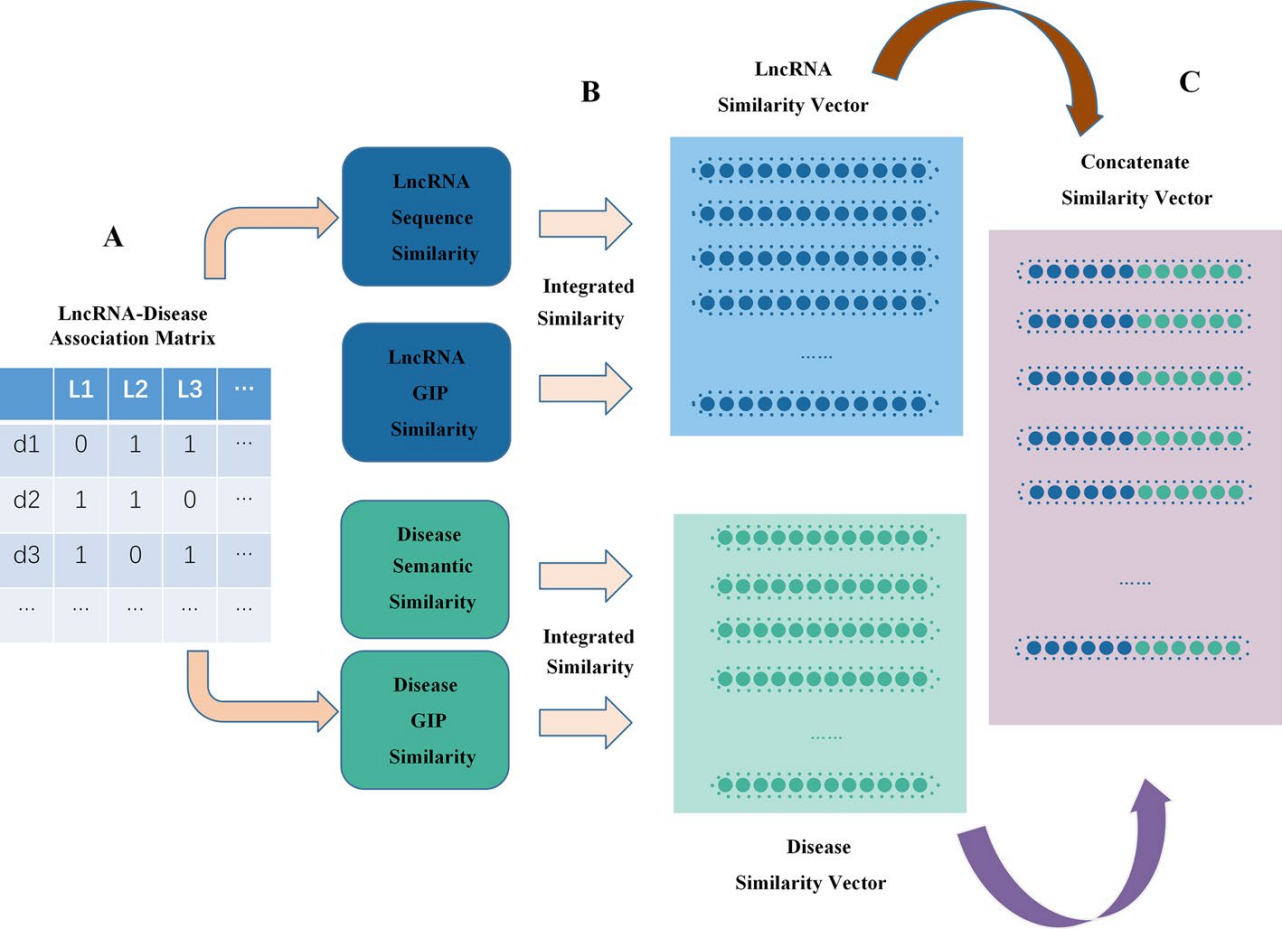
Flowchart of processing similarity features for lncRNAs and diseases

According to the sorted dataset, the interaction information between diseases and lncRNAs was constructed into a matrix $A \in R^{nd \times nl}$, where the columns represent lncRNAs and the rows represent disease. If there was an experimentally verified lncRNA-disease association, the value of A in the matrix was set to 1. Otherwise, the value was set to 0, as shown in Fig. 5A.

#### LncRNA sequence similarity

An increasing number of studies have shown that similar pathologies between two different diseases may be linked with two similar lncRNAs. Therefore, one of the important characteristics of lncRNA-disease association prediction is the similarity between different lncRNAs. Between any two strings, the Levenshtein distance is the minimum cost required for a single word of one string to be converted to the other string after insertion, deletion, or replacement. To investigate the deeper similarity between lncRNAs, we used the Levenshtein distance to calculate the similarity between two lncRNAs. We set the editing cost as 2, and the cost for deletion and insertion as 1. The similarity between the *i*th lncRNA and the *j*th lncRNA is$L_{sim}(l_i,l_j) \in R^{nl \times nl}$, and it can be calculated as follows:

3 $$\begin{aligned} L_{sim}=1- \frac{x}{len(l_i)+len(l_j)} \end{aligned}$$

where *x* represents the minimum cost required to convert one lncRNA sequence into another and *len* represents the sequence length of lncRNA.

#### Disease semantic similarity

In 2010, Schlicker et al. found that the more similar the disease phenotype was, the more similar the gene dysfunction [42]. Gene Ontology annotations provide a way to obtain the semantic similarity of genes [43]. Thus, some researchers employ directed acyclic graphs (*DAGs*) to represent diseases. Additionally, the Jaccard correlation coefficient has been used to calculate the functional similarity of diseases. We applied DAGs to this study to calculate semantic similarity scores for diseases. Let $D_{sim} \in R^{nd \times nd}$ be the disease similarity between the ith disease and the *j*th disease. It can be calculated as follows:

4 $$\begin{aligned} D_{sim}(d_i,d_j)= \frac{\sum _{x\in G_{d_i} \cap G_{d_j}} ( SVD_i(x)+SVD_j(x) )}{ \sum _{x \in G_{d_i} SVD_i(x) } + \sum _{x \in G_{d_j} SVD_j(x) }} \end{aligned}$$

where $G_{d_i}$ represents disease $d_i$ in *DAGs* , $G_{d_j}$represents $d_j$ disease in *DAGs* . Compare disease *i* and disease *j*, $SVD_i(x)$ denotes the disease semantic value of $x \in G_{d_i}$ , and $SVD_j(x)$ denotes the disease semantic value of $x \in G_{d_j}$ .We can calculate the semantic value of a disease *d* by using the following equation:

5 $$\begin{aligned} SVD(x)= {\left\{ \begin{array}{ll} \text{ max } \{\mu \cdot SVD(d^\prime )\}, \text{ if } \quad x \ne d \\ 1, \qquad \quad \qquad \quad \qquad \text{ Otherwise } \end{array}\right. } \end{aligned}$$

where $d^\prime$ $\in$ children of *d*, and $\mu$ represents the factor of semantic contribution. According to previous research, we set it to 0.5 [44].

#### Gaussian interaction profile kernel similarity

Similar lncRNAs may be associated with different diseases that have similar pathological characteristics, and vice versa. Based on this assumption, the kernel similarity between lncRNAs and diseases can be calculated by the Gaussian interaction profile (GIP). The GIP kernel similarities were computed based on the lncRNA-disease interaction matrix obtained from the LncRNADisease dataset. The GIP similarities $GKL(l_i,l_j)$ of lncRNAs can be computed as follows:

6 $$\begin{aligned} GKL(l_i,l_j) = \text{ exp }(-\lambda ||A(l_i)-A(l_j)||^2) \end{aligned}$$

where $A(l_i)$ and $A(l_j)$ represent the *i*th and *j*th columns information in the association matrix *A*. Let $\lambda$ be a parameter that can control the width of the kernel boundary and is represented by the average number of diseases associated with each lncRNA, which is defined as follows:

7 $$\begin{aligned} \lambda = \frac{1}{\frac{1}{nl} \sum _{i=1}^{nl} ||A(l_i)||^2} \end{aligned}$$

where *nl* denotes the number of lncRNAs.

Similarly, we can obtain the GIP kernel similarity of disease $d_i$ and disease $d_j$ as follows:

8 $$\begin{aligned} GKD(d_i,d_j) = \text{ exp }(-\lambda ||A(d_i)-A(d_j)||^2) \end{aligned}$$

where $A(d_i)$ and $A(d_j)$ denote the *i*th and *j*th rows information in the lncRNA-disease association matrix *A*. Let $\lambda$ be a parameter that can control the width of the kernel boundary and is represented by the average number of lncRNAs associated with each disease, which can be calculated as follows:

9 $$\begin{aligned} \lambda = \frac{1}{\frac{1}{nl} \sum _{i=1}^{nd} ||A(d_i)||^2} \end{aligned}$$

where *nd* denotes the number of diseases.

#### Integrated similarity

From the above,the lncRNAs sequence similarity, the semantic similarity of diseases, and the GIP kernel similarity of lncRNAs and diseases were gathered. We obtained the integrated similarity of lncRNA (*Ls*) and integrated similarity of diseases (*Ds*), (Fig. 5B), and the calculation formula is shown as follows:

10 $$\begin{aligned} Ls(l_i,i_j)&= \frac{L_{sim}(l_i,l_j)+GKL(l_i,l_j)}{2} \end{aligned}$$

11 $$\begin{aligned} Ds(d_i,d_j)&= \frac{D_{sim}(d_i,d_j)+GKD(d_i,d_j)}{2} \end{aligned}$$

The disease similarity vector for disease $d_i$ contains the similarity values of all other diseases to $d_i$. Additionally, the lncRNA similarity vector for lncRNA $l_i$ includes the similarity values of all other lncRNAs to $l_i$. Therefore, we concatenated these similarity vectors for the corresponding lncRNA-disease pair to generate large eigenvectors of size $nd+nl$, where the number of diseases and lncRNAs was *nd* and *nl*, as shown in Fig. 5C. There were $nd \times nl$ samples altogether, each corresponding to a lncRNA-disease pair.

### BiGAN

In 2018, Chen et al. proposed using linear-based principal component analysis (PCA) to obtain the traits of GIP kernel similarity [45]. However, the potential lncRNA-disease correlation features were difficult to mine. As a nonlinear generalization of PCA, an auto-encoder is an unsupervised neural network model that mainly includes an encoder and decoder. This special neural network has two advantages in dealing with the features of lncRNA-disease associations [46]. One is that auto-encoders are good at learning biological patterns that are annotated. Second, they can automatically recognize the comprehensive similarity characteristics of lncRNAs and diseases, eliminate noise, and reduce dimensions. This can solve the problem that features extracted from large datasets may produce considerable noise. To further study the model of unsupervised learning, we developed a novel generative adversarial network model inspired by the auto-encoder.

#### The main framework of the BiGAN

**Fig. 6:**
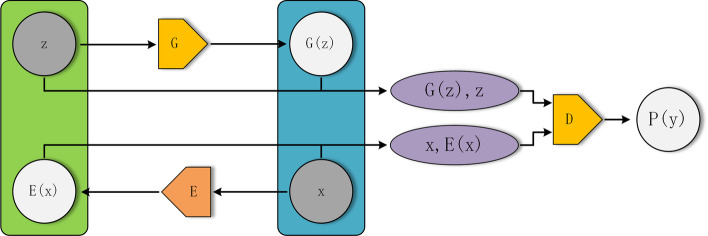
The main framework of BiGAN

In this study, we propose using the bidirectional generative adversarial network(BiGAN) model to complete the task of predicting the association of lncRNA-disease pairs. BiGAN consists of an encoder, a generator, and a discriminator, the main framework of which is shown in Fig. 6. The BiGAN encoder can map the original data point *x* to the latent representation *z*. The BiGAN generator will capture the feature in the latent space to generate a new lncRNA-disease association. The BiGAN discriminator not only discriminates in the traditional data space (*x* versus *G*(*z*)), but also discriminates in the joint data and latent space ((*x*, *E*(*x*)) versus (*G*(*z*), *z*)). The latent component is both an encoder output *E*(*x*) and a generator input *z*.

We can clearly see that the encoder and the generator cannot “communicate” with each other directly. However, the encoder and generator will learn to reverse each other through the joint probability distribution. In other words, *E*(*G*(*z*)) and *G*(*E*(*x*)) can be computed to fool the BiGAN discriminator. In our model, an encoder $E:\Omega _X \rightarrow \Omega _Z$ and a generator $G: \Omega _Z \rightarrow \Omega _X$ are trained at the same time. The BiGAN encoder includes a distribution $P_E(Z|X) = \sigma (Z-E(X))$ mapping data points *x* into a latent feature space of the generator. The BiGAN generator includes a distribution $Q_G(X|Z) = \sigma (X-G(Z))$ extracting randomly sampled noise from the encoder to generat new lncRNA-disease associations. The discriminator will take input from the latent space in to predict the distribution of $P_D(Y|X,Z)$, where the value of *Y* is equal to 0 if *X* is from the output of generator ($G(z),z\sim p_z$), and the value of *Y* is 1 if *X* is sampled from the encoder data distribution $p_x$. Thus, we can define a minimax objective to replace the BiGAN training objective.

12 $$\begin{aligned} \mathop {min}\limits _{G,E} \mathop {max}\limits _{D} V(D,E,G) \end{aligned}$$

where *V*(*D*, *E*, *G*) can be computed based on the following formulas:

13 $$\begin{aligned} V(D,E,G)&= {E}_{X \sim p_X} [logD(X,E(X))] + {E}_{Z \sim p_Z}[log(1-D(G(Z),Z))] \end{aligned}$$

14 $$\begin{aligned} logD(X,E(X))&= {E}_{Z \sim p_E(\cdot |X)} [logD(X,Z)] \end{aligned}$$

15 $$\begin{aligned} log(1-D(G(Z),Z))&= {E}_{X \sim p_G(\cdot |Z)} [log(1-D(X,Z))] \end{aligned}$$

In contrast to other advanced unsupervised computing models, the BiGAN can learn the gradient information perfectly, so as to ensure the correct weight allocation.

#### More details of the encoder, generator, and discriminator

**Fig. 7:**
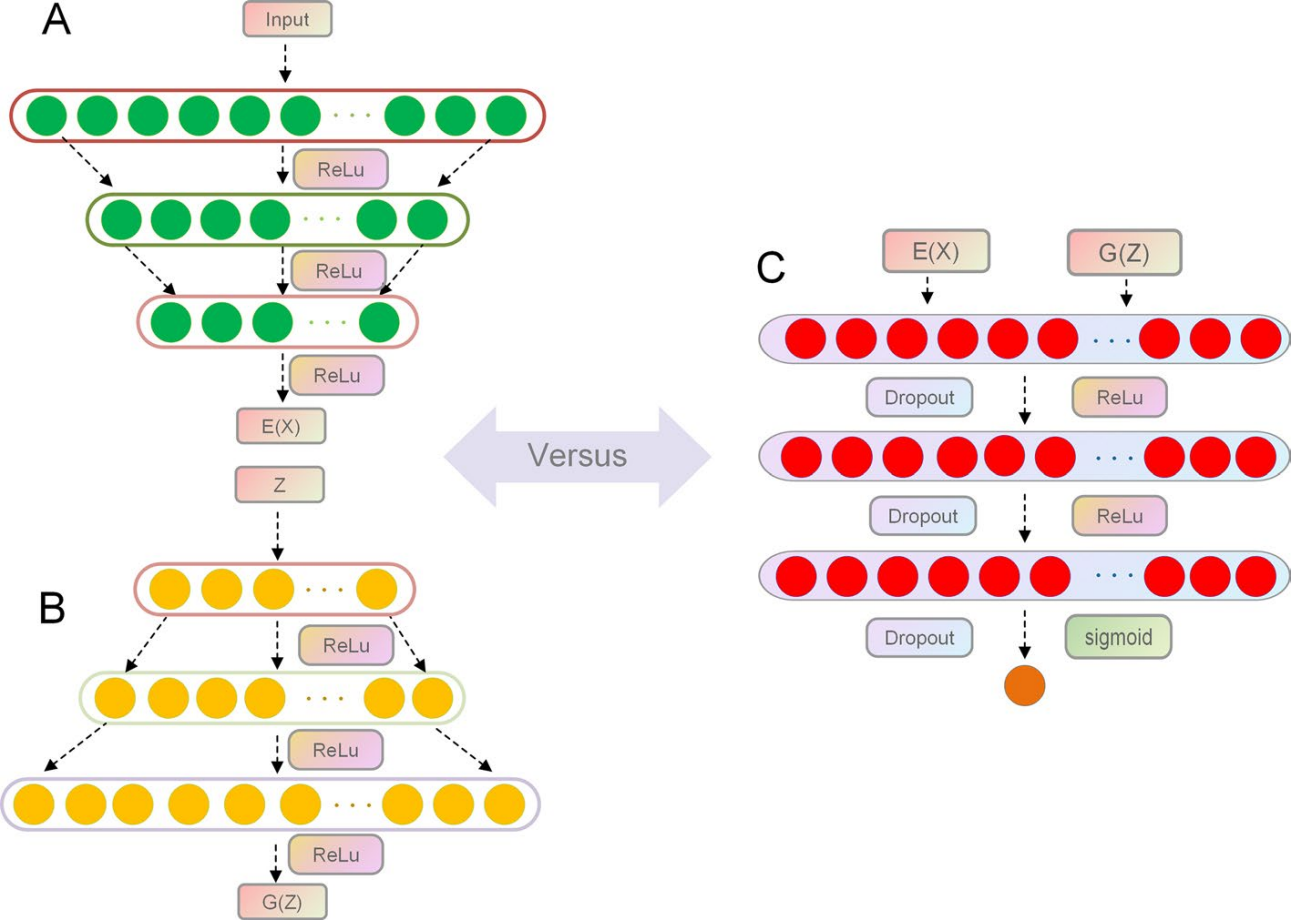
The structure of encoder, generator, and discriminator

*Encoder* In the similarity eigenvectors, each lncRNA contains the similarity information and position information of all other lncRNAs. Likewise, each disease contains information about the similarity and position of all the other diseases. As mentioned above, the BiGAN encoder is one of the two parts of an auto-encoder. The main functions of the encoder are to compress data, eliminate noise, and learn the features of the latent space. We take the similarity feature vectors of the samples as input so that the encoder can fully learn the parameters of the similarity vectors. In this way, the encoder can effectively map the data points into the latent feature space. The structure of BiGAN encoder is shown in Fig. 7A. The encoder is composed of three fully connected layers of the neural network. We can compute the output of each layer with the following formula:

16 $$\begin{aligned} E(x) = W^Ex+b^E \end{aligned}$$

where *x* denotes the similarity features of lncRNA-disease pairs. $W^E$ and $b^E$ represent the encoder weights and bias, respectively.

The dimension of the similarity eigenvectors between the lncRNA and disease will be compressed into a low-dimensional vector after passing through each layer in the encoder. A trained encoder can predict the feature representations of data by capturing semantic attributes. The dense information of compressed low-dimensional vectors is more conducive to learning the mapping relationship of the latent space. To mine the representation of latent space more effectively, we decided to set the number of neurons in the final layer to 100. We employed ReLU as the activation function in the BiGAN model, and it can be defined as follows:

17 $$\begin{aligned} ReLU(y) = {\left\{ \begin{array}{ll} y\quad y\ge 0 \\ 0\quad y<0 \end{array}\right. } \end{aligned}$$

In addition, the encoder will randomly sample noise *z* in distribution $P_E(Z|X) = \sigma (Z-E(X))$ and output latent features *E*(*x*) during training. Ultimately, we can obtain many data pairs (*x*, *E*(*x*)).

*Generator* In most generative adversarial network(GAN) models, the role of the generator is to learn the features of the original data and generate new data based on the learned characteristics. However, in the BiGAN model, the generator takes randomly sampled noise as input. As shown in Fig. 7B, the generator is similar to the encoder in that it has the same network structure. The output of the generator is calculated as follows:

18 $$\begin{aligned} G(z) = W^Gz+b^G \end{aligned}$$

where *z* is the feature of the latent space. $W^G$ and $b^G$ denote the weights and bias of the generator, respectively.

However, each layer in the generator increases the dimension of the potential representation and the final output dimension is the same as the original similarity feature vector dimension. Next, the representation with noise is decoded by the generator, and new lncRNA-disease associations are generated. Then, we can obtain a series of data pairs(G(z),z).

*Discriminator* The two data pairs mentioned above are taken as inputs to fool the discriminator. The discriminator discrimines whether the input data are real. If the discriminator thinks the data pairs come from the encoder, will be set as 1. If the discriminator thinks data pairs come from the generator, it will be set as 0. The structure of the discriminator is shown in Fig. 7C, where the sigmoid function is defined as follows:

19 $$\begin{aligned} sigmoid(\theta ) = \frac{1}{1-e^{(-\theta )}} \end{aligned}$$

where $\theta$ is the input of the sigmoid function.

The BiGAN encoder has a strong representation learning ability to learn the latent association between lncRNAs and diseases. The BiGAN generator will extract the features of the joint data and latent space to generate new lncRNA—disease associations. Finally, $z=E(G(z))$ and $x=G(E(x))$ are determined through a union probability distribution to arrive at a bidirectional structure. And you can see the concrete proof in the study of Jeff et al. According to our experiment, the BiGAN is an unsupervised feature learning model with strong robustness and representational learning ability. Compared with other computing models, the BiGAN performs remarkably well.

## Data Availability

All the data used are collected from the public datasets below. The LncRNAdisease database can be downloaded from http://www.cuilab.cn/lncrnadisease. The Lnc2Cancer database can be downloaded from http://bio-bigdata.hrbmu.edu.cn/lnc2cancer/download.html. The MNDR database can be download from http://www.rna-society.org/mndr/download.html. The source code is available at https://github.com/TomasYang001/BiGAN-lncRNA-disease-associations-prediction.git

## Abbreviations

BiGAN: Bidirectional generative adversarial network
LRLS: Laplacian regularized least square method
LNCSIM: Functional similarity models of lncRNAs
DAGs: Directed acyclic graphs
TPR: True positive rate
FPR: False positive rate
ROC: Receiver operating characteristic
AUC: Areas under ROC curve
AUPR: Areas under precision-recall curve
